# Fecal Microbiome Alteration May Be a Potential Marker for Gastric Cancer

**DOI:** 10.1155/2020/3461315

**Published:** 2020-09-15

**Authors:** Juan Wu, Cong Zhang, Shuo Xu, Chunjie Xiang, Ruiping Wang, Dongqing Yang, Bin Lu, Liyun Shi, Ruimin Tong, Yuhao Teng, Wei Dong, Junfeng Zhang

**Affiliations:** ^1^School of Medicine & Holistic Integrative Medicine, Nanjing University of Chinese Medicine, Nanjing, China; ^2^Department of Oncology, Jiangsu Province Hospital of Traditional Chinese Medicine, Nanjing, China; ^3^Department of Oncology, Yangzhong People's Hospital, Yangzhong, China; ^4^Jiangsu Collaborative Innovation Center Medicine (TCM) Prevention and Treatment of Tumor, Nanjing University of Chinese Medicine, Nanjing, China

## Abstract

Although intestinal microbial dysbiosis was confirmed to be associated with many chronic diseases and health status through complicated interaction with the host, the effect on gastric cancer was less studied. In this study, we sequenced the 16S rRNA and 18S rRNA genes of fecal bacteria and fungi, respectively, in 134 gastric cancer patients and 58 healthy controls matched by age and gender. Propensity score matching (PSM) was adopted for adjusting diet habits and lifestyle, and 44 patients and 44 healthy controls (matching population) were enrolled. Serum antibody to *H. pylori* and metabolites of the matching population were detected. The positive rates of antibody to *H. pylori* between the patients and the control group did not reach the statistical difference. LEfSe analysis indicated that bacteria were more stable than fungi when adjusting diet and lifestyle. *Veillonella*, *Megasphaera*, and *Prevotella 7* genus and *Streptococcus salivarius* subsp. *Salivarius*, *Bifidobacterium dentium*, and *Lactobacillus salivarius* species in bacteria were related to the risk of gastric cancer and showed a good diagnostic value in distinguishing the patients from healthy controls. *Streptococcus mitis* showed a risk effect for gastric cancer; however, the effect turned into be protective after PSM. Serum L-alanine, L-threonine, and methionol were positively associated with *Veillonella* and *Streptococcus* and several fungi genus. Overall, our findings indicated that fecal microbiome constitution alteration may be associated with gastric cancer through influencing the amino acid metabolism.

## 1. Introduction

Gastric cancer (GC) ranked the third most commonly diagnosed cancer and the second most common cause of cancer death in 2015 in China [1]. Epidemiologists found that salty and smoked food intake, lack of fresh fruits and vegetable consumption, cigarette and alcohol consumption, and *H. pylori* infection were the main risk factors of GC. Among these, *H. pylori* infection was the only confirmed bacterium in GC and regarded as I carcinogenic factor by IARC [2]. Besides the *H. pylori*, other bacteria were also confirmed to survive in the hostile environment of the stomach [3]. The high infection rate of *H. pylori* [4] and low incident rate of GC among the infected population may indicate the more complex ecosystem in the stomach.

In general, the microbiota is thought to reside in the human body from birth and mature in the first year of life. Recently, some researchers argued the time of colonization could be even earlier because they found the placenta is not sterile in healthy pregnant women [5]. The gastrointestinal tract, from the oral cavity through the esophagus and to the rectum, is a complex and dynamic ecosystem, and the diversity and abundance of microbes change markedly. The colon and distal gut have the largest ecosystem in the body (about 10^12^ per gram of contents), and this ecosystem was regarded as a metabolic organ [6]. They can produce short-chain fatty acids (SCFAs) by soluble dietary fiber fermentation, and SCFAs (especially acetate, propionate, and butyrate) are important modulators not only maintaining energy homeostasis of the gut epithelial cell but also involved in metabolic syndrome and its associated diseases such as diabetes and obesity [7].

Recently, gut microbiomes become a rapidly advancing field in human cancers, which shapes a microenvironment for host cells that can either prevent or promote cancer formation. For instance, *Fusobacterium nucleatum*, which originated from the oral cavity, could potentiate the carcinogenesis of colorectal cancer by interaction with other microorganisms in the intestine, such as *Peptostreptococcus* spp. and *Leptotrichia* spp., and the mechanism which involved the activation of Wnt target genes increased the secretion of proinflammatory cytokines and evading anticancer immunes response [8, 9]. Furthermore, other tumors, such as breast cancer, pancreatic cancer, and prostate cancer, were also found to be related to the diversity and community of fecal microbiomes [10–12]. The intestinal flora could participate in the metabolic biosynthesis and immune response and thus provide the possibility in the GC carcinogenesis. Related research is relatively scarce. A study from Shanxi Province, China, disclosed the bacteria community alteration in fecal may be associated with GC [13]. Our previous study showed that the lifestyle contributed greatly to the GC risk and gender difference [14], since diet and lifestyle were the pivotal factors in shaping the gut microbes. Propensity score matching (PSM) methods could eliminate the influence of lifestyle which may attenuate the reliability and relevance of the fecal bacteria and the risk of GC.

In contrast to the bacteria, the altered components of fungi were less studied in human health and disease condition. The first aim of our study is to profile the fecal microbiomes, both bacteria and fungi, in GC patients. Unlike the genetic and epigenetic elements, the constitution of the gut microbiome could be improved or changed by probiotic, prebiotic, and symbiotic. Thus, we tried to provide a new biomarker and/or preventive target for GC. Additionally, to understand the effect of microbiota more deeply, we tried to explore the association of the fecal microbiota with the metabolic features in GC patients.

## 2. Methods

### 2.1. Study Participants and Sample Collection

A total of 192 individuals, 134 GC patients and 58 healthy controls, were enrolled in this study from January 2015 to January 2017 in Jiangsu Province Hospital of TCM. GC patients were diagnosed by endoscopy combined with pathological biopsy. The exclusion criteria for patients included (1) chemotherapy or biotherapy prior to stool sample collection, (2) diagnosis of other malignancies within 5 years from the time of recruitment, and (3) antibiotics or probiotics for the last 4 weeks. The healthy control subjects with no intestinal diseases were recruited from the individuals attending annual health check at the same hospital and matched to the GC patients by sex and gender. The inclusion criteria for the controls included the following: (1) having not used antibiotics, probiotics, and acid blocker for the last 4 weeks; (2) without any malignant tumor; (3) without type 2 diabetes and other metabolic diseases; and (4) digestive system disease-related serum markers (i.e., carbohydrate antigen (CA)19-9; carcinoembryonic antigen, CEA; and alpha-fetoprotein, AFP) within the normal range. Information on dyspeptic symptoms and intestinal disease status were self-reported and were collected by a questionnaire. All the subjects were with Han nationality. This study was approved by the institutional ethics review committee of Jiangsu Province Hospital of TCM (grant no. 2015 NL-016-01), and all study participants signed written informed consent before the interview and sample collection. All patients provided fecal samples and peripheral blood samples. Fresh stool samples were collected in a clean environment and placed in a sterile sampling tube within 2 hours after excretion. Samples were immediately preserved at -80°C until assay.

### 2.2. Propensity Score Matching

Propensity score matching (PSM) methods were adopted to further match the GC patients and healthy controls through age, gender, smoking, drinking status, and some diet habits (eating speed, salty, fried foods, and fruits consumption) [15]. The value of calipers was limited to 0.1, logistic regression was used to obtain the propensity score (PS), and a ratio of 1 : 1 was used to obtain the matching population.

### 2.3. Serum *H. pylori* Antibody Detection

The serum *H. pylori* antibody was detected by western blot using a typing detection kit for antibody to *H. pylori* (Shenzhen Blot Biotech, China).

### 2.4. Serum Sample Preparation and GC/MS Analysis

The serum sample was taken from -80°C and thawed at 4°C. Thawed serum was placed at room temperature for 30 min and vortexed for 5 s. 50 *μ*L serum was mixed with 25 *μ*L internal standard (1 g/L, heptadecanoic acid-methanol) and vortexed for 15 s. Subsequently, 150 *μ*L of methanol was added to the tube, vortexed for 15 s, and centrifuged at 13 000 rpm at 4°C for 10 min. 185 *μ*L of supernatant was centrifuged and dried in a vacuum at room temperature for 2 h. 25 *μ*L of methoxyamine-pyridine (20 g/L) was subsequently added and vortexed for 15 s, and the mixture was incubated in 80°C for 15 min, then cooled in room temperature. 25 *μ*L of *N*-methyl-*N*-trimethylsilyltrifluoracetamid was added, vortexed for 15 s, and incubated at 80°C for 15 min; then, the resultant was cooled in room temperature and centrifuged at 13,000 rpm at 4°C for 10 min. 50 *μ*L of supernatant was added into a 1.5 mL centrifugal tube. The reference sample was used to ensure the stability and reliability of the metabonomics, and blank samples are used to eliminate the background noise produced during the sample preparation and GC/MS analysis.

The derivatized serum samples were analyzed on PerkinElmer Clarus 680 gas chromatography-tandem with AxION-iQT mass spectrometer (PerkinElmer, US). An HP-5MS Quartz capillary column (30 m × 0.25 mm × 0.25 *μ*m, Agilent, US) was utilized to separate the derivatives.

Helium was used as the carrier gas with a 20 : 1 shunt ratio at a flow rate of 1 mL/min through the column. The injection port temperature is 200°C, the detection temperature is 250°C, and the injection volume was 1 *μ*L. The temperature program is as follows: the initial temperature was held at 50°C for 4 min, ramped to 140°C at a rate of 10°C/min for 4 min, and then ramped to 170°C at a rate of 2°C/min for 6 min. The mass spectrometry conditions are as follows: the temperature of ion source (electron impact) was set at 230°C, collision energy was 70 eV, and interface temperature of GC/MS was 230°C. Mass data were acquired in a full-scan mode (m/z 40-400). All samples were analyzed in a random sequence.

### 2.5. GC/MS Data Processing and Structural Identification of Metabolites

The acquired GC/MS data were uploaded to the XCMS online platform (http://xcmsonline.scripps.edu) to extract chromatographic peak, compute peak area integral, correct retention time, deduct the impurity peak and solvent peak, etc. TIC (total ions chromatogram) and preprocessed data of GC patients and healthy controls were obtained and imported into the SIMCA software (version 14.1, Sweden), where principal component analysis (PCA) and orthogonal partial least squares-discriminant analysis (OPLS-DA) were performed. The OPLS-DA model was validated by 200 times permutation analysis. Variable importance in the projection (VIP) was obtained from the OPLS-DA model. A differential normalized peak area between the two groups was compared by two-tailed Student's test (if the distribution was normal), otherwise by Mann–Whitney *U* test on the XCMS online platform; meanwhile, the fold change (FC) was calculated. Receiver operating characteristic curve (ROC), the area under the curve (AUC), 95% CI, specificity, and sensitivity were analyzed by the “Biomarker discovery” module on MetaboAnalyst 4.0 platform (http://www.metaboanalyst.ca/faces/home.xhtml).

The METLIN online database was used to identify metabolites preliminarily by charge-to-mass ration and retention time of pretreated samples. The metabolites were confirmed by the KEGG (http://www.genome.jp/kegg/kegg2.html), HMDB (http://www.hmdb.ca/metabolites), and PubChem (https://pubchem.ncbi.nlm.nih.gov) online databases.

### 2.6. Gene Amplification

Total microbial DNA was isolated from fecal samples by E.Z.N.A.^®^ Soil DNA Kit (Omega Bio-Tek, Norcross, GA, US) according to the manufacturer's protocols. Bacterial 16S rRNA V3-V5 region gene was chosen and amplified by PCR. The forward prime was 341F 5′-barcode-CCTAYGGGRBGCASCAG-3′, and the reverse primer was 806R 5′-GGACTACNNGGGTATCTAAT-3′; the ITS1-2 regions in the fungal 18S rRNA gene were amplified using the forward primer ITS1F 5′-barcode-CTTGGTCATTTAGAGGAAGTAA-3′ and reverse primer ITS2R 5′-GCTGCGTTCTTCATCGATGC-3′. The PCR conditions were the same as our previous study [16]. The amplified DNA was then purified and quantified by QuantiFluor™-ST Assay Kit (Promega, US).

### 2.7. Gene Sequencing and Data Analyses

Purified amplicons were equally pooled, and the barcode was added. The profiling was carried out on an Illumina Hiseq platform. Low-quality sequencing reads were filtered and removed by QIIME (version 1.17) [16]. UPARSE (version 7.1) was used to cluster the filtered sequences into an operational taxonomic unit (OTUS) based on 97% similarity. Chimeric sequences were identified and deleted by the UCHIME algorithm. Each OTU was aligned to the SILVA database with a confidence threshold of 70% to perform the taxonomic analysis. Mothur (version v.1.30.1) was used to analyze the alpha diversities (i.e., diversity within samples) of gut microbial communities. Linear discriminant analysis (LDA) effect size (Lefse) was used to analyze the differential abundance of six levels (phylum, class, order, family, genus, and species) between two groups.

When we did the Pearson correlation between gut microbiota and serum metabolites, we limited the bacterial taxa (phylum to genus) with mean relative abundance ≥0.1%, and we did not limit the mean relative abundance of the fungi taxa for the overall relative low abundance of fungi. AUC (Area Under Curve) of ROC (receiver operating characteristic curve) was calculated for each significant taxon. Chi-square or Fisher's exact test was used to frequency table data, and the independent *t*-test or the nonparametric rank test was for continuous data.

### 2.8. Statistical Analyses

The significant level of all analysis was 0.05, and all statistical tests were two-sided. The analyses were carried out using R (version 3.4.2) and SPSS for Windows (version 23.0).

## 3. Results

### 3.1. The Common Clinical Characteristics

The characteristics of the overall participants (134 vs. 58) and matched by PSM (44 vs. 44) are listed in Table 1. In the matching population, besides the age and gender, other potential confounders were also balanced between the two groups (all *P* values >0.05), compared to the original population. The positive rate of antibody to *H. pylori* did not reach the statistical differences between the two groups.

The alpha diversities of bacteria and fungi between the GC and control groups were compared both in the original and matching population (i.e., ACE, Chao, Shannon, and Simpson index), and we did not find any difference in the bacteria group (all *P* values >0.05). In the original population, the Chao index of fungi in the control group was higher than that of the GC group (*P* = 0.034); however, in the matching group, the difference disappeared (*P* = 0.346), see Supplementary Table 1.

### 3.2. Community Structure of Gut Flora

**Figure 1:**
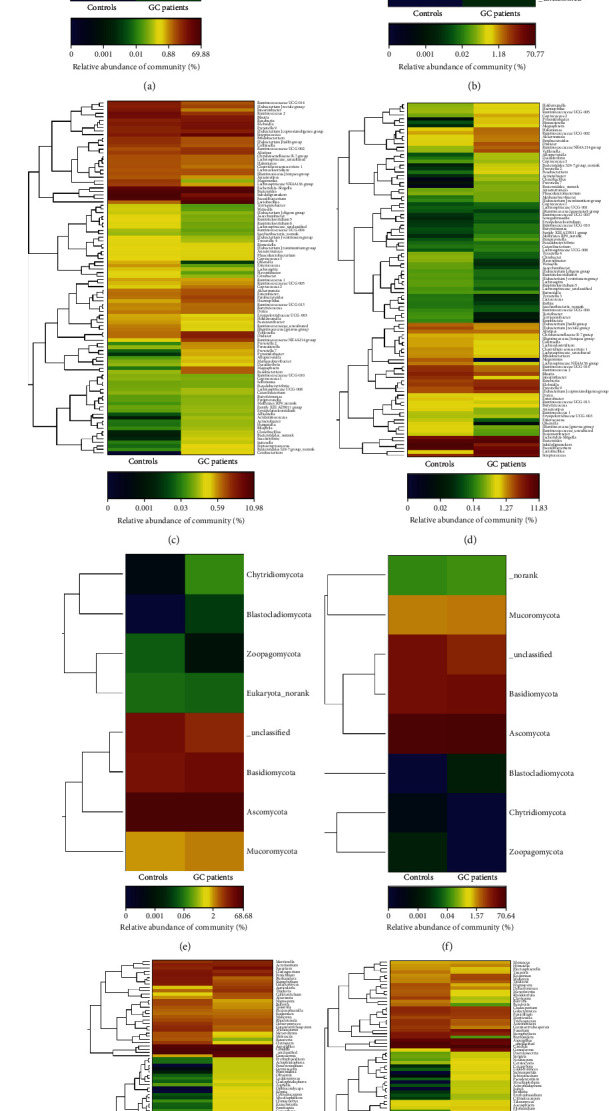
The community structure of gut microbiota in the population. (a) presented the bacterial community at the phylum level for original population, (b) presented the bacterial community at the phylum level for matching population, (c) presented the bacterial community of top 50 gener of original population, (d) presented the bacterial community of top 50 genera of matching population, (e) presented the fungal community at the phylum level of original population, (f) presented the fungal community at the phylum level of matching population, (g) presented the fungal community of the top 50 genera for the original population, and (h) presented the fungal community of the top 50 genera of matching population.

The community structure of fecal bacteria and fungi in phylum and genus levels (top 50) were shown in Figure 1. In the phylum level, fecal bacteria were dominated by *Firmicutes*, *Proteobacteria*, and *Bacteroidetes* (Figure 1(a) for the original population and Figure 1(b) for the matching population) and fecal fungi were dominated by *Basidiomycota* and *Ascomycota* (Figure 1(e) for the original population and Figure 1(f) for the matching population). Compared to the GC group, the relative abundance of *Cyanobacteria* phylum was reduced in the control group (*P* = 0.028) in the original population, however, no significant differences of fungi phylum levels in matching population were found. In the genus level, the relative abundance of the top 50 bacteria genus is shown in Figures 1(c) and 1(d) (Figure 1(c) for the original population and Figure 1(d) for the matching population, respectively) and the top 50 fungi genus is shown in Figures 1(g) and 1(h) (Figure 1(g) for the original population and Figure 1(h) for the matching population, respectively).

### 3.3. Effect of Lifestyle on the Potential Diagnostic Value of Fecal Flora

**Figure 2:**
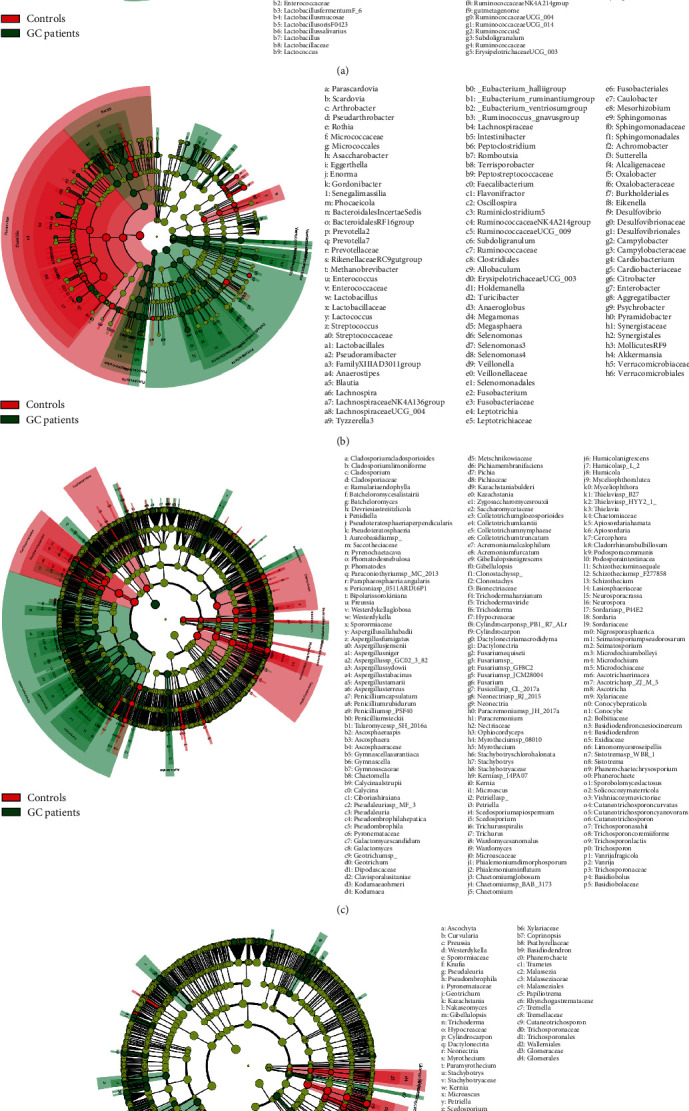
LEfSe analysis of gut microbiome between GC patients and healthy controls. (a) showed the different bacterial taxa of the original population, (b) showed the different bacterial taxa of the matching population, (c) showed the different fungal taxa of the original population, and (d) showed the different fungal taxa of the matching population.

LEfSe analysis was conducted to compare the bacteria and fungi taxa differences from phylum to species between the GC and control groups. The community difference distribution of fecal fungi was dramatically changed before and after adjusting lifestyle and diet habits (see Figure 2(c) for the original population and Figure 2(d) for matching population, respectively). On the other hand, this change was not obvious in the bacteria community (see Figures 2(a) and 2(b)). To explore the potential diagnostic microbiota biomarkers, we conducted ROC analysis and obtained AUC of each different taxa.

For fecal bacteria, the specific taxa associated with GC in the original population and matching population are listed in Supplementary Table 2 and Supplementary Table 3, respectively. In the original population, the relative abundance of *Veillonella* genus in GC was significantly higher than that in the control group (*P* < 0.001), and the AUC = 0.837 (95% CI: 0.772-0.902); in addition, the AUC of four species (*Streptococcus mitis*, *Bifidobacterium dentium Bd1*, *Streptococcus salivarius* subsp. *salivarius*, and *Bifidobacterium dentium*) and two genera (*Megasphaera* and *Atopobium*) were more than 0.7, and all these taxa were associated with an increased risk for GC. In the matching population, the trend of *Veillonella* genus was consistent with that in the original population, and the AUC = 0.855 (95% CI: 0.773-0.937). In addition, the AUC of four species (*Bifidobacterium dentium*, *Streptococcus salivarius* subsp. *salivarius*, *Streptococcus mitis*, and *Lactobacillus salivarius*) and three genera (*Megasphaera*, *Prevotella 7*, *Desulfovibrio*) were more than 0.7; all those taxa had an increased risk for GC, except for the *Streptococcus mitis*. To acquire the more stable and relevant diagnostical microbiota biomarkers, we selected the common different bacteria taxa both in the original and matching population, and three genera (*Veillonella*, *Megasphaera*, and *Prevotella 7*) and four species (*Streptococcus mitis*, *Streptococcus salivarius subsp. salivarius*, *Bifidobacterium dentium*, and *Lactobacillus salivarius*) were obtained (see Table 2). Unlike other taxa, *Streptococcus mitis* in the matching population was associated with a decreased risk for GC; it may indicate the risk effect of this species depends on the external environment elements.

For fecal fungi, the specific taxa associated with GC in the original population and matching population is listed in Supplementary Table 4 and Supplementary Table 5, respectively. In the original population, the AUC of nine taxa reached 0.6 and higher; they were the *Auriculariales* order, *Pyronemataceae*, *Stachybotryaceae*, *Lasiosphaeriaceae* family, *Humicola*, *Kazachstania* genera, *Fusarium* sp., *Petriella* sp., and *Aspergillus terreus* species. All those taxa were related to an increased risk for GC. In the matching population, the AUC of *Hypocreaceae*, *Stachybotryaceae*, *Lasiosphaeriaceae*, *Sporormiaceae* family, *Humicola*, *Chaetomium* genera, and *Petriella* sp. reached 0.6 and higher.

### 3.4. Cluster Analysis of Fecal Flora

**Figure 3:**
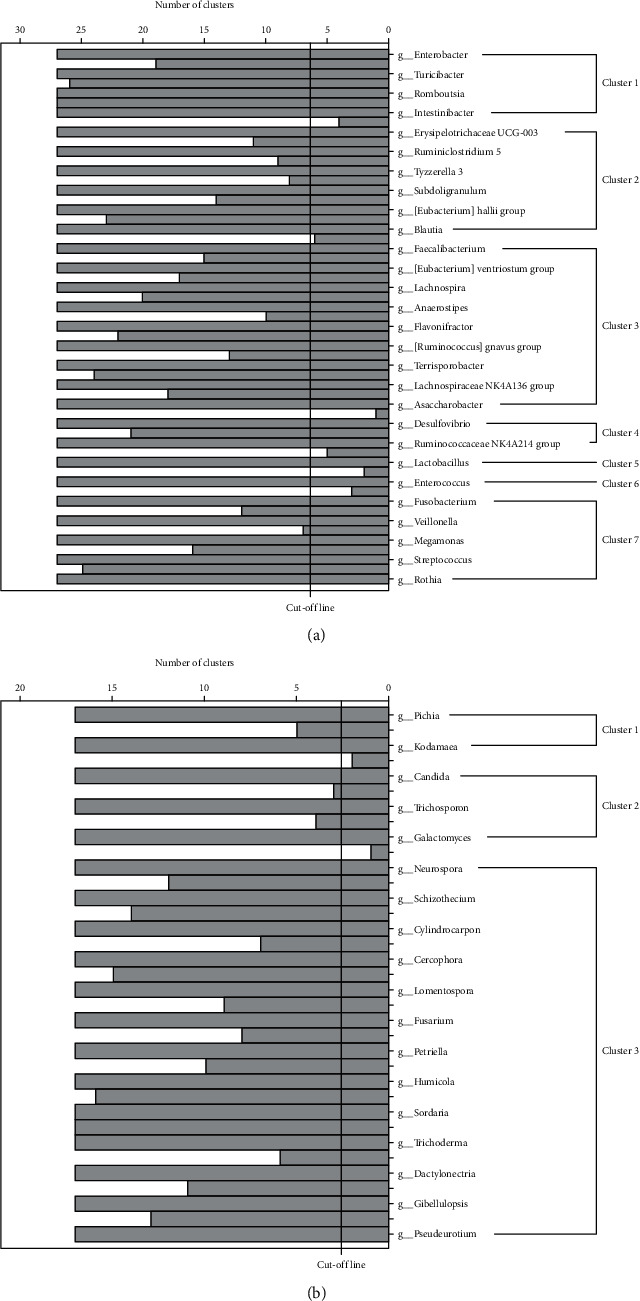
Hierarchical ward-linkage clustering based on the Pearson correlation coefficient of the fold change of each relative abundance. (a) demonstrated the seven clusters for gut bacteria; (b) demonstrated the three clusters for gut fungi.

**Figure 4:**
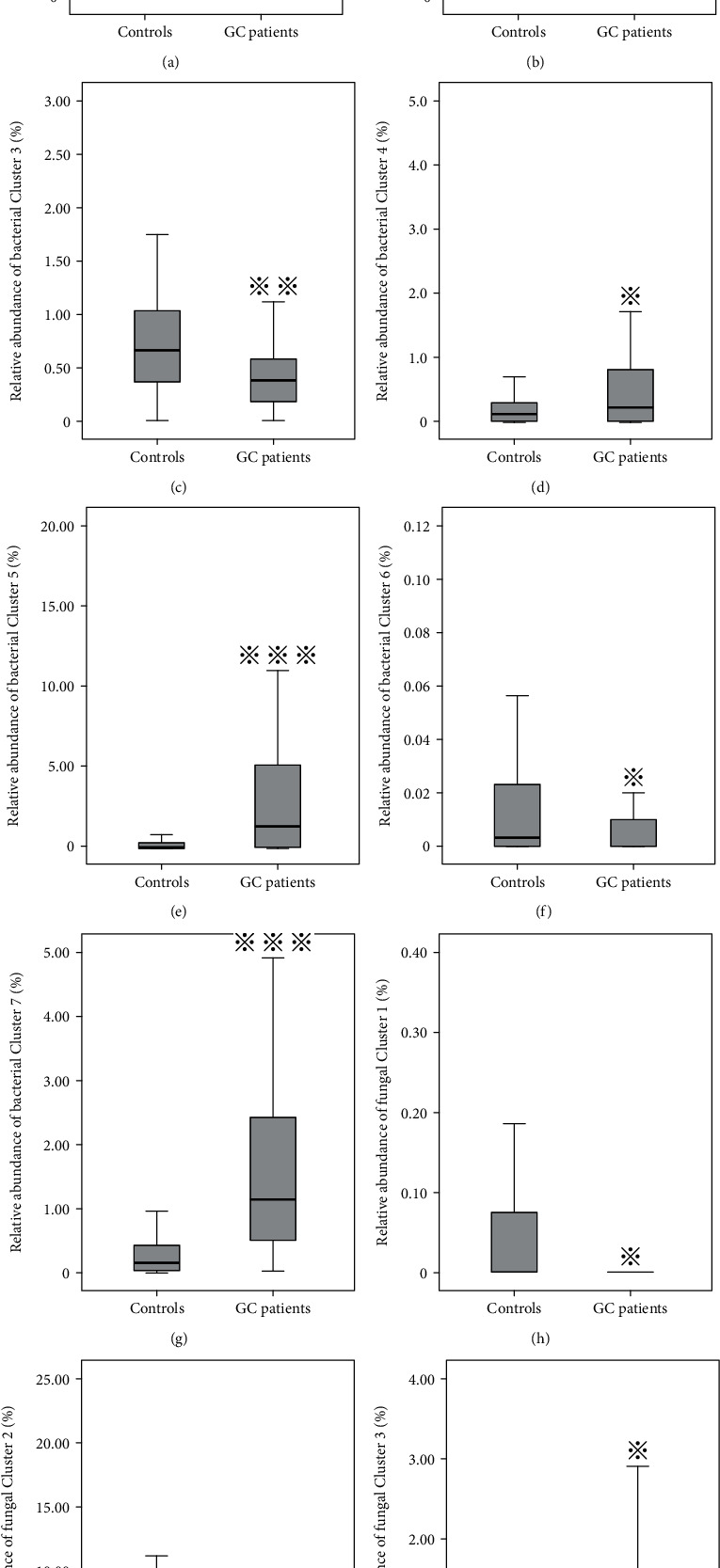
Boxplots of relative abundance of the seven gut bacteria clusters (a to g) and three gut fungi clusters (h to j) between the GC patients and healthy controls. ^∗^ indicated the *P* value less than 0.05, ^∗∗^ indicated the *P* value less than 0.01, and ^∗∗∗^ indicated the *P* value less than 0.001.

The fecal microbiota is a complex ecosystem, and the community structure may be more informative than the individual taxa abundance difference. We clustered the differential 28 genera of bacteria and 18 genera of fungi in the matching population into seven and three clusters, respectively, by hierarchical ward-linkage clustering based on the Pearson correlation coefficient of the fold change of each relative abundance (Figure 3) [16]. We then compared the mean relative abundance of each cluster between the GC and control groups (Figure 4). For fecal bacteria, the relative abundances of Cluster 4, Cluster 5, and Cluster 7 in the GC group were higher than that in the control group and may have a potential risk effect for GC; on the contrary, Cluster 2, Cluster 3, and Cluster 6 in the GC group were lower than that in the control group. For fecal fungi, the relative abundances of Cluster 2 in the control group were higher, and the relative abundance of Cluster 3 in the GC group was higher.

### 3.5. Correlation between Serum Metabolites and Fecal Flora

**Figure 5:**
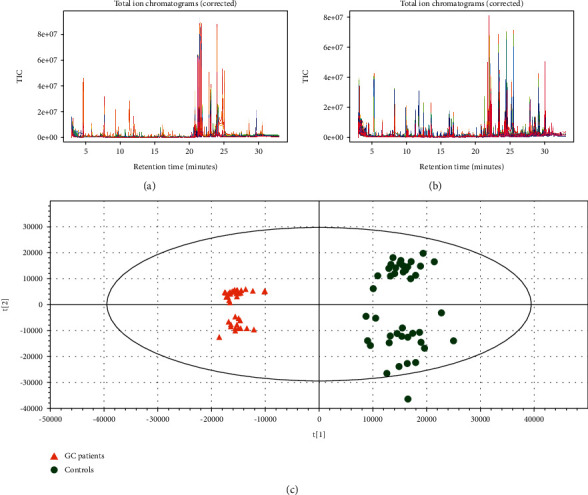
TIC (total ions chromatogram) chromatographs from healthy controls (a) and GC patients (b); OPLS-DA (orthogonal partial least squares-discriminant analysis) between GC patients and healthy controls (c).

**Figure 6:**
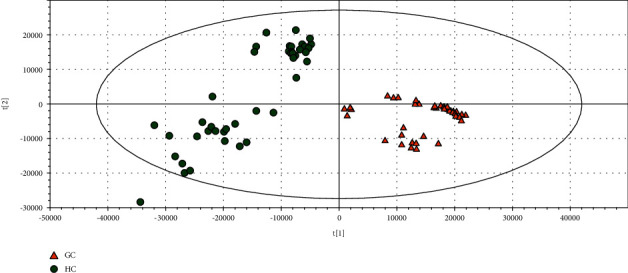
Principal component analysis (PCA) of serum metabolite difference between GC patients and healthy controls.

**Figure 7:**
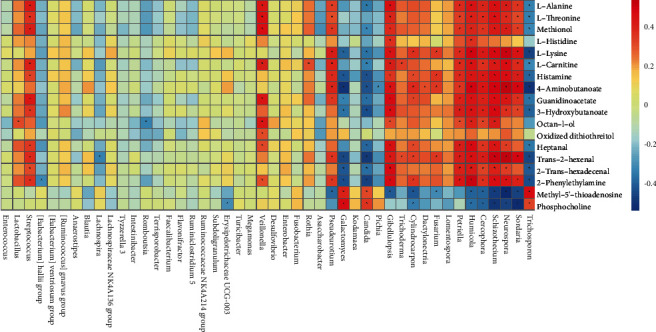
Pearson correlation of different gut microbiota (both bacteria and fungi) with serum metabolite, selecting the relative abundance of bacteria >0.1%.

GC/MS-based metabolomics was performed to profile the serum metabolite of GC patients and healthy controls. TIC chromatographs from healthy controls and GC patients are shown in Figures 5(a) and 5(b), respectively. Furthermore, we conducted OPLS-DA (4 PCs, *R*^2^*X* = 0.81, *R*^2^*Y* = 0.97, *Q*^2^ = 0.967), and a dramatic difference between the GC patients and healthy controls was observed (Figure 5(c)). PCA was also conducted to illustrate the metabolic difference between the two groups (12 PCs, *R*^2^*X* = 0.951, *Q*^2^ = 0.892), and the results showed a good performance in distinguishing the two groups (Figure 6). After the structure identification of metabolites, we chose the mean relative abundance of bacteria genus more than 0.1% and performed the association of gut microbiota (both bacteria and fungi) with the serum metabolite. The results are shown in Figure 7. Serum amino acid (L-alanine, L-threonine, methionol, L-carnitine, guanidinoacetate), heptanal, and phenylethylamine were positively related to bacteria *Streptococcus* and *Veillonella* genus. Three serum amino acids (L-alanine, L-threonine, methionol) were also correlated with fungi *Pseudeurotium*, *Gibellulopsis*, *Petriella*, *Humicola*, *Cercophora*, *Schizothecium*, *Neurospora*, and *Sordaria* genus.

## 4. Discussion

In this study, we identified the potential association of fecal microbiota, both bacteria and fungi, with the occurrence of GC and the host serum metabolites. As the largest habitat, intestine microbes were represented as the most important “organ” that not only of the digestion but in the host immune and metabolic system [17]. Thus, increasing evidence showed that dysbiosis of intestinal microbiomes could influence the initiation and development of systemic disease beyond the gut and even the effect of immunotherapy [18]. Fecal specimens represent a conveniently accessible and noninvasive source for investigating the gut microbiota composition. Thus, fecal samples were used to evaluate whether the fecal microbiome can be used as a biomarker affecting the risk of gastric cancer. To exclude the confounding elements, we matched the GC patients and healthy controls with the diet and some lifestyle habits. Compared to the bacteria, the fungi community was more susceptible to environmental elements. This result was consistent with previous findings. David et al. found that animal- or plant-based diets have a different influence on fungi species; they could increase the abundance of *Candida* and *Penicillium* species, respectively [19]. The rapid alteration of fungi was also confirmed in the murine gut [20]. This may be explained that the abundance of bacteria is larger than fungi; thus, their communities are more robust than that of fungi.

The genus *Lactobacillus* is taxonomically complex and contains over 170 species. Although they are part of the normal commensal microbes in human gastrointestinal and extensively used in commercial products and dairy food, such as yogurt and cheese, they can also be human pathogens occasionally. Some *Lactobacillus* species were isolated from a variety of human infections, and in most cases, malignancies or gastrointestinal disorders were the main underlying diseases [21, 22]. Our results indicated that *Lactobacillus salivarius* relative abundance was higher in GC cases than in healthy controls, and this tendency is not influenced by environmental factors. This indicates the *Lactobacillus* species may be related to the malignancies, represented by GC. This is consistent with our gastric mucosal specimen data, which suggests that there may be a correlation between the intestinal flora and the gastric mucosal flora [23].

We also found some common oral community members (i.e., genus *Veillonella*, *Streptococcus mitis*, *Streptococcus salivarius*, and *Fusobacterium*) tend to be related to the risk of GC. *Streptococcus mitis* is a pioneer colonizer in the human oral cavity and could produce hydrogen peroxide (H_2_O_2_) to inhibit the growth of downstream bacteria sensitive to hyperoxic stress [24]. In addition, the reactive oxygen species (ROS), such as H_2_O_2_, are known to be related to mutagenesis, tumorigenesis, and aging through damaging various biological macromolecules. As early colonizers and most predominant oral bacteria, the species of *Veillonella* genus can coaggregate with the initial colonizer such as *Streptococcus mitis*. *Veillonella*, a bridging species, not only provide food and site to downstream pathogens, such as *Fusobacterium nucleatum* and *Porphyromonas gingivalis*, but also protect them from oxygen stress and support the survival. Except for periodontal diseases, *Fusobacterium nucleatum* and *Porphyromonas gingivalis* were regarded as the carcinogenic organisms of colorectal cancer and esophageal cancer, respectively [8, 25]. Understanding the interaction between the later and pioneer colonizers would lead to the development in disease prevention for GC.

Through the serum metabolite analysis, we found bacteria *Streptococcus* and *Veillonella* were both positively correlated with serum amino acid (L-alanine, L-threonine, methionol, L-carnitine, guanidinoacetate), heptanal, and phenylethylamine. Fungi *Pseudeurotium*, *Gibellulopsis*, *Petriella*, *Humicola*, *Cercophora*, *Schizothecium*, *Neurospora*, and *Sordaria* were also correlated with these three serum amino acids (i.e., L-alanine, L-threonine, methionol). It may indicate metabolic of the amino acid are shared cross the bacteria and eukaryotes. Amino acid homeostasis is essential for the balance of cellular amino acid pools in the microbiota. Except for the metabolic prerequisite for the microbe's growth, the balance could also ensure the host amino acid homeostasis. L-alanine, for instance, has a great abundance in cellular concentration, only second to glutamate and aspartate, and participates in amino acid biosynthetic and catabolic pathways and thereby connecting crucial metabolic networks [26, 27]. L-threonine was thought to generate SCFAs, acetate, butyrate, and propionate and plays a prominent role in maintaining proper intestinal function and integrity [28]. Abubucker et al. identified several biosynthesis genes for L-threonine metabolism in human gut microbiota using metagenomic data [29], and our association data also confirm the relationship between this amino acid and gut microbiota.

Although the gut is dominated by bacteria, other microbes, such as fungi, archaea, and phages, also reside in the gut. The number of fungi in the gut is far lower than that of bacteria, but the fungal cells are much bigger and more complex [30]. The fungi dysbiosis has also been reported to be accompanied by diseases such as inflammatory bowel diseases [31]. The Candida-derived prostaglandin E2 (PGE2) can reach the lungs and promote allergic inflammation through acting on lung macrophages [32]. Intestinal fungi could influence immunity at distant body sites through both productions of fungal metabolites and interaction with immune cells [33]. We described both fecal bacteria and fungi community alteration associated with GC and serum metabolites, and it may provide some clues for the further investigation of the complex interaction of gut microbiota and the host.

There are some limitations in this study. First, in terms of the selection of the control group, we did not use gastroscopy to exclude patients with gastric diseases, who have no obvious symptoms of dyspepsia. Since the control group came from the normal physical examination population in the hospital, in addition to using the questionnaire to collect gastric symptoms and disease status information, we also checked the patient's digestive system disease-related serum markers (i.e., carbohydrate antigen (CA)19-9; carcinoembryonic antigen, CEA; and alpha-fetoprotein, AFP) and selected those individuals with normal indicators. Second, we used the serum sample to test the antibody against the *H. pylori* instead of ^13^C-urea breath test, rapid urease test, or histological staining of *H. pylori*, which do not always reflect current infection. Third, self-reports of lifestyle habits (eating speed, taste, and so on) may be biased. Large sample research with multicenter cooperation is needed in follow-up research.

## 5. Conclusion

Taken together, the findings of our study suggest that fecal bacteria and fungi community alteration may be related to the risk of GC with the same *H. pylori* infection status. The relative abundance of *Veillonella*, *Megasphaera*, and *Prevotella 7* genus and *Streptococcus salivarius* subsp., *Salivarius*, *Bifidobacterium dentium*, and *Lactobacillus salivarius* species in bacteria increased in the GC patients and showed a good diagnostic value in distinguishing the patients from healthy controls. Serum L-alanine, L-threonine, and methionol were positively associated with *Veillonella* and *Streptococcus* and several fungi genus. The fecal microbiome community change may be a new biomarker and/or preventive target for GC. Our results should be considered as preliminary due to the small sample size. Thus, further studies in a larger population with different ethnicities were encouraged.

## Figures and Tables

**Table 1:** Clinical characteristics of GC patients and healthy controls.

Variables		Original population				Matching population			
		Cases (*n* = 134)	Controls (*n* = 58)	*t*/*χ*^2^	*P*	Cases (*n* = 44)	Controls (*n* = 44)	*t*/*χ*^2^	*P*
Age (year)	Mean ± SD	64.5 ± 9.2	65.9 ± 9.4	0.930	0.353	64.8 ± 9.8	63.8 ± 9.7	0.439	0.662
Gender	Male	109 (81.3%)	44 (75.9%)	0.751	0.386	30 (68.2%)	30 (68.2%)	0.000	1.000
	Female	25 (18.7%)	14 (24.1%)			14 (31.8%)	14 (31.8%)		
Eating speed	<10 min	65 (48.5%)	13 (22.4%)	11.426	0.001	14 (31.8%)	13 (29.5%)	0.053	0.817
	≥10 min	69 (51.5%)	45 (77.6%)			30 (68.2%)	31 (70.5%)		
Salty foods	Yes	60 (44.8%)	9 (15.5%)	15.052	<0.001	11 (25.0%)	9 (20.5%)	0.259	0.611
	No	74 (55.2%)	49 (84.5%)			33 (75.0%)	35 (79.5%)		
Fried foods	Yes	109 (81.3%)	41 (70.7%)	2.688	0.101	31 (70.5%)	30 (68.2%)	0.053	0.817
	No	25 (18.7%)	17 (29.3%)			13 (29.5%)	14 (31.8%)		
Fruits	Yes	51 (38.1%)	46 (79.3%)	27.555	<0.001	29 (65.9%)	32 (72.7%)	0.481	0.488
	No	83 (61.9%)	12 (20.7%)			15 (34.1%)	12 (27.3%)		
Smoking	Yes	86 (64.2%)	14 (24.1%)	26.005	<0.001	14 (31.8%)	14 (31.8%)	0.000	1.000
	No	48 (35.8%)	44 (75.9%)			30 (68.2%)	30 (68.2%)		
Drinking	Yes	81 (60.4%)	22 (37.9%)	8.253	0.004	17 (38.6%)	20 (45.5%)	0.420	0.517
	No	53 (39.6%)	36 (62.1%)			27 (61.4%)	24 (54.5%)		

**Table 2:** The GC-risk related gut microbiota identified by the ROC analysis.

The different bacterial taxa	The original population				The matching population			
	Relative abundance (%)		*Z*(*P*)	AUC (95% CI)	Relative abundance (%)		*Z*(*P*)	AUC (95% CI)
	Cases (*n* = 134)	Controls (*n* = 58)			Cases (*n* = 44)	Controls (*n* = 44)		
g_Veillonella	2.25 ± 3.57	0.78 ± 3.76	7.409 (0.000)	0.837 (0.772, 0.902)	2.18 ± 3.97	0.37 ± 1.45	5.741 (0.000)	0.855 (0.773, 0.937)
s_Streptococcus mitis	0.37 ± 0.74	0.26 ± 1.13	5.583 (0.000)	0.754 (0.679, 0.829)	0.29 ± 0.52	0.33 ± 1.29	3.541 (0.000)	0.719 (0.610, 0.828)
g_Megasphaera	0.94 ± 4.29	0.03 ± 0.11	5.458 (0.000)	0.743 (0.670, 0.815)	0.73 ± 3.10	0.04 ± 0.13	4.097 (0.000)	0.745 (0.640, 0.849)
s_Streptococcus salivarius subsp. salivarius	2.77 ± 3.29	1.26 ± 4.14	5.231 (0.000)	0.738 (0.663, 0.813)	2.79 ± 3.54	1.50 ± 4.72	3.843 (0.000)	0.738 (0.633, 0.842)
s_Bifidobacterium dentium	0.08 ± 0.23	0.02 ± 0.08	4.787 (0.000)	0.705 (0.629, 0.781)	0.06 ± 0.10	0.01 ± 0.06	4.238 (0.000)	0.741 (0.635, 0.848)
s_Lactobacillus salivarius	0.92 ± 1.88	0.08 ± 0.24	4.418 (0.000)	0.695 (0.621, 0.769)	0.84 ± 1.77	0.06 ± 0.21	3.452 (0.001)	0.705 (0.594, 0.815)
g_Prevotella 7	0.44 ± 1.73	0.02 ± 0.10	4.591 (0.000)	0.694 (0.618, 0.771)	0.30 ± 0.73	0.01 ± 0.07	4.201 (0.000)	0.739 (0.635, 0.844)

## Data Availability

The data used to support the findings of this study are available from the corresponding author upon request.

## Abbreviations

GC: Gastric cancer
HP: *Helicobacter pylori*
OTUs: Operational taxonomic units
PSM: Propensity score matching
SCFAs: Short-chain fatty acids
PCA: Principal component analysis
OPLS-DA: Orthogonal partial least squares-discriminant analysis
FC: Fold change
ROC: Receiver operating characteristic curve
AUC: The area under the curve
LDA: Linear discriminant analysis
CAGs: Coabundance groups.

## Supplementary Materials

Supplementary 1 Table 1: The alpha diversity of gut microbiome in GC patients and healthy controls [Median(Q25,Q75)].

Supplementary 2 Table 2: ROC analysis of the different gut bacteria between the GC patients and controls in the original population.

Supplementary 3 Table 3: ROC analysis of the different gut bacteria between the GC patients and controls in the matching population.

Supplementary 4 Table 4: ROC analysis of the different gut fungi between the GC patients and controls in the original population.

Supplementary 5 Table 5: ROC analysis of the different gut fungi between the GC patients and controls in the matching population.

## References

[1] Chen W., Zheng R., Baade P. D. (2016). Cancer statistics in China, 2015. *CA: a Cancer Journal for Clinicians*.

[2] Parkin D. M. (2006). The global health burden of infection-associated cancers in the year 2002. *International Journal of Cancer*.

[3] Lam S. Y., Yu J., Wong S. H., Peppelenbosch M. P., Fuhler G. M. (2017). The gastrointestinal microbiota and its role in oncogenesis. *Best Practice & Research Clinical Gastroenterology*.

[4] Hooi J. K. Y., Lai W. Y., Ng W. K. (2017). Global prevalence of Helicobacter pylori infection: systematic review and meta-analysis. *Gastroenterology*.

[5] Aagaard K., Ma J., Antony K. M., Ganu R., Petrosino J., Versalovic J. (2014). The placenta harbors a unique microbiome. *Science Translational Medicine*.

[6] Sender R., Fuchs S., Milo R. (2016). Revised estimates for the number of human and bacteria cells in the body. *PLoS Biology*.

[7] Hu J., Lin S., Zheng B., Cheung P. C. K. (2018). Short-chain fatty acids in control of energy metabolism. *Critical Reviews in Food Science and Nutrition*.

[8] Brennan C. A., Garrett W. S. (2019). Fusobacterium nucleatum - symbiont, opportunist and oncobacterium. *Nature Reviews Microbiology*.

[9] Proença M. A., Biselli J. M., Succi M. (2018). Relationship betweenFusobacterium nucleatum, inflammatory mediators and microRNAs in colorectal carcinogenesis. *World Journal of Gastroenterology*.

[10] Michaud D. S., Izard J. (2014). Microbiota, oral microbiome, and pancreatic cancer. *Cancer Journal*.

[11] Goedert J. J., Jones G., Hua X. (2015). Investigation of the association between the fecal microbiota and breast cancer in postmenopausal women: a population-based case-control pilot study. *Journal of the National Cancer Institute*.

[12] Alanee S., el-Zawahry A., Dynda D. (2019). A prospective study to examine the association of the urinary and fecal microbiota with prostate cancer diagnosis after transrectal biopsy of the prostate using 16sRNA gene analysis. *The Prostate*.

[13] Qi Y.-F., Sun J.-N., Ren L.-F. (2019). Intestinal microbiota is altered in patients with gastric cancer from Shanxi Province, China. *Digestive Diseases and Sciences*.

[14] Zhang J., Zhan Z., Wu J. (2013). Association among polymorphisms in EGFR gene exons, lifestyle and risk of gastric cancer with gender differences in Chinese Han subjects. *PLoS One*.

[15] Newgard C. D., Hedges J. R., Arthur M., Mullins R. J. (2004). Advanced statistics: the propensity score--a method for estimating treatment effect in observational research. *Academic Emergency Medicine*.

[16] Wu J., Xu S., Xiang C. (2018). Tongue coating microbiota community and risk effect on gastric cancer. *Journal of Cancer*.

[17] Gu Y., Liu C., Zheng N., Jia W., Zhang W., Li H. (2019). Metabolic and gut microbial characterization of obesity-prone mice under a high-fat diet. *Journal of Proteome Research*.

[18] McQuade J. L., Daniel C. R., Helmink B. A., Wargo J. A. (2019). Modulating the microbiome to improve therapeutic response in cancer. *The Lancet Oncology*.

[19] David L. A., Maurice C. F., Carmody R. N. (2014). Diet rapidly and reproducibly alters the human gut microbiome. *Nature*.

[20] Dollive S., Chen Y. Y., Grunberg S. (2013). Fungi of the murine gut: episodic variation and proliferation during antibiotic treatment. *PLoS One*.

[21] Salminen M. K., Rautelin H., Tynkkynen S. (2004). Lactobacillus bacteremia, clinical significance, and patient outcome, with special focus on probiotic L. rhamnosus GG. *Clinical Infectious Diseases : an Official Publication of the Infectious Diseases Society of America*.

[22] Salminen M. K., Rautelin H., Tynkkynen S. (2006). Lactobacillus bacteremia, species identification, and antimicrobial susceptibility of 85 blood isolates. *Clinical Infectious Diseases : an Official Publication of the Infectious Diseases Society of America*.

[23] Wu Z. F., Zou K., Wu G. N. (2020). A comparison of tumor-associated and non-tumor-associated gastric microbiota in gastric cancer patients. *Digestive Diseases and Sciences*.

[24] Zhou P., Li X., Huang I. H., Qi F. (2017). Veillonella catalase protects the growth of Fusobacterium nucleatum in microaerophilic and Streptococcus gordonii-resident environments. *Applied and Environmental Microbiology*.

[25] Peters B. A., Wu J., Pei Z. (2017). Oral microbiome composition reflects prospective risk for esophageal cancers. *Cancer Research*.

[26] Metges C. C. (2000). Contribution of microbial amino acids to amino acid homeostasis of the host. *The Journal of Nutrition*.

[27] Peña-Soler E., Fernandez F. J., López-Estepa M. (2014). Structural analysis and mutant growth properties reveal distinctive enzymatic and cellular roles for the three major L-alanine transaminases of *Escherichia coli*. *PLoS One*.

[28] Gaifem J., Gonçalves L. G., Dinis-Oliveira R. J. (2018). L-threonine supplementation during colitis onset delays disease recovery. *Frontiers in Physiology*.

[29] Abubucker S., Segata N., Goll J. (2012). Metabolic reconstruction for metagenomic data and its application to the human microbiome. *PLoS Computational Biology*.

[30] Zuo T., Wong S. H., Cheung C. P. (2018). Gut fungal dysbiosis correlates with reduced efficacy of fecal microbiota transplantation in Clostridium difficile infection. *Nature Communications*.

[31] Zuo T., Ng S. C. (2018). The gut microbiota in the pathogenesis and therapeutics of inflammatory bowel disease. *Frontiers in Microbiology*.

[32] Kim Y. G., Udayanga K. G. S., Totsuka N., Weinberg J. B., Núñez G., Shibuya A. (2014). Gut dysbiosis promotes M2 macrophage polarization and allergic airway inflammation via fungi-induced PGE2. *Cell Host & Microbe*.

[33] Richard M. L., Sokol H. (2019). The gut mycobiota: insights into analysis, environmental interactions and role in gastrointestinal diseases. *Nature Reviews Gastroenterology & Hepatology*.

