# Supplementary material for: Fecal Microbiome Alteration May Be a Potential Marker for Gastric Cancer
Source: Dis Markers. 2020 Sep 15;2020:3461315. doi: 10.1155/2020/3461315 (PMC7519184; doi:10.1155/2020/3461315)
Supplement: Supplementary 1 — Table 1: The alpha diversity of gut microbiome in GC patients and healthy controls [Median(Q25,Q75)]. [file 3461315.f1.docx]

**Table S1.** The alpha diversity of gut microbiome in GC patients and healthy controls [Median(Q25,Q75)]

| Variable | | Before matching | | | | After matching | | | |
| --- | --- | --- | --- | --- | --- | --- | --- | --- | --- |
| Domain | Diversity index | Cases(n=134) | Controls (n=58) | Z | P | Cases (n=44) | Controls (n=44) | Z | P |
| Bacteria | Ace | 786.0(647.2,929.2) | 823.7(687.5,921.4) | 0.885 | 0.376 | 829.7(681.9,965.9) | 822.1(700.1,916.7) | 0.134 | 0.894 |
|  | Chao | 727.0(591.9,844.4) | 744.4(612.8,847.2) | 0.441 | 0.659 | 749.0(607.9,847.0) | 752.3(618.7,841.1) | 0.142 | 0.887 |
|  | Shannon | 3.781(3.445,4.122) | 3.766(3.315,4.045) | 0.854 | 0.393 | 3.796(3.538,4.226) | 3.766(3.336,4.050) | 1.093 | 0.274 |
|  | Simpson | 0.056(0.039,0.093) | 0.063(0.048,0.096) | 1.261 | 0.207 | 0.059(0.039,0.078) | 0.064(0.049,0.102) | 1.085 | 0.278 |
| Fungi | Ace | 195.2(149.4,267.1) | 235.8(162.3,285.2) | 1.853 | 0.064 | 211.4(164.8,282.6) | 243.5(170.5,284.3) | 0.743 | 0.458 |
|  | Chao | 190.4(148.3,261.3) | 244.7(164.2,293.8) | 2.123 | 0.034 | 207.5(163.3,295.9) | 252.6(169.3,294.3) | 0.943 | 0.346 |
|  | Shannon | 3.326(2.339,3.957) | 3.296(2.755,3.758) | 0.173 | 0.863 | 3.345(2.479,3.943) | 3.243(2.753,3.825) | 0.392 | 0.695 |
|  | Simpson | 0.084(0.038,0.237) | 0.083(0.048,0.137) | 0.133 | 0.894 | 0.082(0.038,0.234) | 0.083(0.049,0.181) | 0.300 | 0.764 |
