# Supplementary material for: Fecal Microbiome Alteration May Be a Potential Marker for Gastric Cancer
Source: Dis Markers. 2020 Sep 15;2020:3461315. doi: 10.1155/2020/3461315 (PMC7519184; doi:10.1155/2020/3461315)
Supplement: Supplementary 2 — Table 2: ROC analysis of the different gut bacteria between the GC patients and controls in the original population. [file 3461315.f2.docx]

**Table S2.** ROC analysis of the different gut bacteria between the GC patients and controls in the original population

| The different bacterial taxa | Relative abundance [(Mean±SD) %] | | *Z*(*P*) | AUC (95%CI) |
| --- | --- | --- | --- | --- |
|  | GC patients (n=134) | Control (n=58) |  |  |
| g__Veillonella | 2.25±3.57 | 0.78±3.76 | 7.409(0.000) | 0.837(0.772,0.902) |
| s__Streptococcus mitis | 0.37±0.74 | 0.26±1.13 | 5.583(0.000) | 0.754(0.679,0.829) |
| g__Megasphaera | 0.94±4.29 | 0.03±0.11 | 5.458(0.000) | 0.743(0.670,0.815) |
| s__Bifidobacterium dentium Bd1 | 0.06±0.18 | 0.01±0.07 | 5.480(0.000) | 0.738(0.665,0.811) |
| s__Streptococcus salivarius subsp. salivarius | 2.77±3.29 | 1.26±4.14 | 5.231(0.000) | 0.738(0.663,0.813) |
| g__Atopobium | 0.04±0.06 | 0.02±0.07 | 4.972(0.000) | 0.725(0.647,0.803) |
| s__Bifidobacterium dentium | 0.08±0.23 | 0.02±0.08 | 4.787(0.000) | 0.705(0.629,0.781) |
| s__Lactobacillus salivarius | 0.92±1.88 | 0.08±0.24 | 4.418(0.000) | 0.695(0.621,0.769) |
| g__Prevotella 7 | 0.44±1.73 | 0.02±0.10 | 4.591(0.000) | 0.694(0.618,0.771) |
| g__Allisonella | 0.12±0.46 | 0.02±0.10 | 4.702(0.000) | 0.691(0.616,0.766) |
| s__Clostridium perfringens B str. ATCC 3626 | 0.15±0.48 | 0.03±0.20 | 4.475(0.000) | 0.683(0.606,0.761) |
| s__Streptococcus anginosus subsp. anginosus | 0.01±0.01 | 0.00±0.00 | 4.778(0.000) | 0.682(0.607,0.757) |
| g__Fusobacterium | 0.93±3.97 | 0.13±0.55 | 3.911(0.000) | 0.676(0.597,0.755) |
| g__Rothia | 0.06±0.08 | 0.10±0.47 | 3.878(0.000) | 0.676(0.589,0.762) |
| s__Streptococcus mutans ATCC 25175 | 0.09±0.22 | 0.01±0.05 | 4.039(0.000) | 0.673(0.595,0.750) |
| g__Desulfovibrio | 0.31±0.67 | 0.12±0.30 | 3.426(0.001) | 0.653(0.570,0.736) |
| g__Anaeroglobus | 0.03±0.18 | 0.00±0.01 | 3.773(0.000) | 0.646(0.566,0.727) |
| g__Gemella | 0.04±0.09 | 0.02±0.06 | 3.227(0.001) | 0.645(0.564,0.726) |
| s__Lactobacillus mucosae | 0.85±2.98 | 0.14±0.68 | 3.291(0.001) | 0.643(0.565,0.722) |
| s__Pyramidobacter piscolens | 0.47±2.42 | 0.01±0.04 | 3.507(0.000) | 0.643(0.564,0.722) |
| s__[Clostridium] clostridioforme 90A3 | 0.29±0.75 | 0.10±0.26 | 3.086(0.002) | 0.640(0.554,0.725) |
| s__Clostridium perfringens CPE str. F4969 | 0.29±1.02 | 0.05±0.27 | 3.235(0.001) | 0.637(0.558,0.717) |
| g__Granulicatella | 0.06±0.09 | 0.06±0.24 | 2.816(0.005) | 0.628(0.543,0.712) |
| g__Actinomyces | 0.05±0.07 | 0.04±0.07 | 2.795(0.005) | 0.627(0.541,0.713) |
| s__Lactobacillus fermentum F-6 | 0.07±0.36 | 0.01±0.03 | 3.160(0.002) | 0.625(0.544,0.706) |
| s__Streptococcus mutans GS-5 | 0.00±0.01 | 0.00±0.00 | 3.284(0.001) | 0.612(0.530,0.693) |
| g__Dialister | 2.35±5.20 | 0.78±1.93 | 2.403(0.016) | 0.609(0.524,0.694) |
| g__Alloprevotella | 0.38±1.68 | 0.06±0.26 | 2.838(0.005) | 0.604(0.521,0.687) |
| g__Ruminococcaceae NK4A214 group | 1.52±3.11 | 0.36±0.78 | 2.247(0.025) | 0.602(0.522,0.682) |
| g__Sutterella | 0.17±0.89 | 0.04±0.09 | 2.277(0.023) | 0.602(0.518,0.686) |
| g__Phascolarctobacterium | 0.30±0.53 | 0.28±1.13 | 2.220(0.026) | 0.601(0.516,0.686) |
| g__Enterobacter | 1.06±4.40 | 0.80±2.98 | 2.223(0.026) | 0.601(0.507,0.695) |
| g__Klebsiella | 3.07±6.44 | 2.62±8.45 | 2.173(0.030) | 0.599(0.511,0.687) |
| p__Cyanobacteria | 0.05±0.21 | 0.01±0.02 | 2.191(0.028) | 0.598(0.513,0.682) |
| s__Streptococcus sobrinus | 0.05±0.15 | 0.00±0.02 | 2.822(0.005) | 0.597(0.515,0.679) |
| g__Parabacteroides | 1.34±1.78 | 0.73±0.88 | 2.121(0.034) | 0.597(0.512,0.681) |
| g__Prevotella 2 | 0.59±2.40 | 0.04±0.18 | 2.320(0.020) | 0.590(0.508,0.673) |
| s__Sphingomonas phyllosphaerae | 0.02±0.14 | 0.00±0.01 | 2.610(0.009) | 0.590(0.506,0.673) |
| g__Achromobacter | 0.00±0.01 | 0.00±0.00 | 2.990(0.003) | 0.589(0.507,0.672) |
| s__Cardiobacterium valvarum | 0.00±0.00 | 0.00±0.00 | 2.862(0.004) | 0.589(0.506,0.673) |
| g__Halomonas | 0.91±4.10 | 1.04±2.53 | 1.987(0.047) | 0.410(0.322,0.497) |
| o__Pseudomonadales | 0.13±1.41 | 0.04±0.18 | 2.069(0.039) | 0.409(0.322,0.496) |
| g__[Ruminococcus] gauvreauii group | 0.05±0.09 | 0.11±0.21 | 2.064(0.039) | 0.407(0.314,0.500) |
| g__Ruminococcaceae UCG-004 | 0.08±0.11 | 0.14±0.23 | 2.179(0.029) | 0.404(0.316,0.492) |
| g__Megamonas | 0.12±0.62 | 1.45±6.61 | 2.654(0.008) | 0.403(0.311,0.494) |
| g__[Ruminococcus] gnavus group | 0.32±0.92 | 0.76±2.19 | 2.369(0.018) | 0.394(0.308,0.480) |
| g__Eggerthella | 0.05±0.14 | 0.08±0.16 | 2.462(0.014) | 0.391(0.304,0.477) |
| g__Turicibacter | 0.03±0.09 | 0.16±0.61 | 2.643(0.008) | 0.389(0.302,0.477) |
| g__Ruminococcus 2 | 1.58±3.08 | 3.94±8.03 | 2.500(0.012) | 0.387(0.297,0.476) |
| g__Lachnospira | 0.14±0.29 | 0.40±1.28 | 2.612(0.009) | 0.381(0.292,0.470) |
| g__Tyzzerella 3 | 0.03±0.12 | 0.11±0.54 | 3.308(0.001) | 0.378(0.288,0.467) |
| g__Ruminococcaceae UCG-014 | 1.03±2.48 | 2.44±5.80 | 2.737(0.006) | 0.377(0.289,0.464) |
| g__Blautia | 2.60±2.32 | 3.73±3.43 | 2.878(0.004) | 0.369(0.285,0.454) |
| g__Lactococcus | 0.03±0.09 | 0.11±0.59 | 2.943(0.003) | 0.367(0.282,0.452) |
| s__Asaccharobacter celatus | 0.09±0.37 | 0.25±0.44 | 3.095(0.002) | 0.365(0.272,0.458) |
| g__Flavonifractor | 0.07±0.15 | 0.29±0.86 | 3.005(0.003) | 0.364(0.280,0.449) |
| g__Faecalibacterium | 5.22±6.88 | 9.29±9.39 | 3.017(0.003) | 0.363(0.275,0.451) |
| s__Enterococcus saccharolyticus subsp. saccharolyticus ATCC 43076 | 0.03±0.14 | 0.17±0.60 | 3.438(0.001) | 0.360(0.271,0.450) |
| g__Fusicatenibacter | 0.28±0.47 | 0.54±0.78 | 3.198(0.001) | 0.355(0.271,0.438) |
| g__Terrisporobacter | 0.06±0.19 | 0.19±0.59 | 3.327(0.001) | 0.353(0.268,0.438) |
| g__[Eubacterium] ventriosum group | 0.13±0.30 | 0.24±0.36 | 3.255(0.001) | 0.352(0.270,0.435) |
| g__Anaerostipes | 0.53±1.16 | 0.99±1.37 | 3.414(0.001) | 0.345(0.258,0.431) |
| g__[Eubacterium] hallii group | 1.14±2.32 | 1.69±1.79 | 3.871(0.000) | 0.324(0.239,0.408) |
| g__Intestinibacter | 0.68±1.31 | 2.79±6.43 | 4.035(0.000) | 0.316(0.234,0.399) |
| g__Erysipelotrichaceae UCG-003 | 0.31±0.66 | 0.46±0.55 | 4.084(0.000) | 0.315(0.234,0.397) |
| s__Gordonibacter pamelaeae | 0.00±0.01 | 0.01±0.03 | 4.678(0.000) | 0.313(0.227,0.399) |
| g__Subdoligranulum | 3.10±5.02 | 10.71±14.06 | 4.155(0.000) | 0.311(0.221,0.401) |
| g__Lachnospiraceae NK4A136 group | 0.49±1.30 | 1.40±2.33 | 4.434(0.000) | 0.298(0.214,0.382) |
| g__Ruminiclostridium 5 | 0.10±0.18 | 0.24±0.31 | 5.052(0.000) | 0.271(0.197,0.344) |
| g__Romboutsia | 0.02±0.07 | 0.16±0.44 | 5.414(0.000) | 0.261(0.181,0.342) |
