# Supplementary material for: Fecal Microbiome Alteration May Be a Potential Marker for Gastric Cancer
Source: Dis Markers. 2020 Sep 15;2020:3461315. doi: 10.1155/2020/3461315 (PMC7519184; doi:10.1155/2020/3461315)
Supplement: Supplementary 3 — Table 3: ROC analysis of the different gut bacteria between the GC patients and controls in the matching population. [file 3461315.f3.docx]

**Table S3.** ROC analysis of the different gut bacteria between the GC patients and controls in the matching population

| The different bacterial taxa | Relative abundance [(Mean±SD)%] | | Z(P) | AUC (95%CI) |
| --- | --- | --- | --- | --- |
|  | GC patients (n=44) | Control (n=44) |  |  |
| g__Veillonella | 2.18±3.97 | 0.37±1.45 | 5.741(0.000) | 0.855(0.773,0.937) |
| g__Megasphaera | 0.73±3.10 | 0.04±0.13 | 4.097(0.000) | 0.745(0.640,0.849) |
| s__Bifidobacterium dentium | 0.06±0.10 | 0.01±0.06 | 4.238(0.000) | 0.741(0.635,0.848) |
| g__Prevotella 7 | 0.30±0.73 | 0.01±0.07 | 4.201(0.000) | 0.739(0.635,0.844) |
| s__Streptococcus salivarius subsp. salivarius | 2.79±3.54 | 1.50±4.72 | 3.843(0.000) | 0.738(0.633,0.842) |
| s__Streptococcus mitis | 0.29±0.52 | 0.33±1.29 | 3.541(0.000) | 0.719(0.610,0.828) |
| g__Desulfovibrio | 0.44±0.62 | 0.13±0.32 | 3.432(0.001) | 0.708(0.599,0.817) |
| s__Lactobacillus salivarius | 0.84±1.77 | 0.06±0.21 | 3.452(0.001) | 0.705(0.594,0.815) |
| s__Streptococcus mutans ATCC 25175 | 0.07±0.12 | 0.01±0.06 | 3.433(0.001) | 0.696(0.584,0.807) |
| s__Streptococcus anginosus subsp. anginosus | 0.00±0.01 | 0.00±0.00 | 3.802(0.000) | 0.690(0.577,0.802) |
| s__Clostridium perfringens B str. ATCC 3626 | 0.18±0.61 | 0.04±0.23 | 3.283(0.001) | 0.682(0.569,0.795) |
| o__Burkholderiales | 1.07±5.39 | 0.13±0.21 | 2.660(0.008) | 0.665(0.551,0.778) |
| g__Fusobacterium | 0.58±1.67 | 0.12±0.56 | 2.650(0.008) | 0.661(0.547,0.776) |
| s__Lactobacillus fermentum F-6 | 0.08±0.44 | 0.00±0.01 | 3.095(0.002) | 0.654(0.539,0.770) |
| g__Anaeroglobus | 0.01±0.05 | 0.00±0.01 | 3.113(0.002) | 0.653(0.537,0.768) |
| g__Rothia | 0.06±0.07 | 0.13±0.54 | 2.412(0.016) | 0.649(0.533,0.764) |
| g__Ruminococcaceae NK4A214 group | 1.69±3.44 | 0.39±0.88 | 2.207(0.027) | 0.636(0.520,0.753) |
| s__Streptococcus mutans GS-5 | 0.00±0.01 | 0.00±0.00 | 2.886(0.004) | 0.628(0.511,0.746) |
| s__Pyramidobacter piscolens | 0.88±3.74 | 0.01±0.04 | 2.321(0.020) | 0.628(0.510,0.745) |
| g__Enterobacter | 0.63±1.27 | 0.91±3.40 | 2.073(0.038) | 0.628(0.509,0.747) |
| s__[Clostridium] clostridioforme 90A3 | 0.32±0.91 | 0.12±0.29 | 2.032(0.042) | 0.625(0.508,0.743) |
| g__Cardiobacterium | 0.00±0.01 | 0.00±0.00 | 2.673(0.008) | 0.623(0.506,0.741) |
| g__Turicibacter | 0.02±0.07 | 0.19±0.70 | 2.207(0.027) | 0.371(0.254,0.488) |
| g__Megamonas | 0.00±0.00 | 1.82±7.55 | 2.379(0.017) | 0.372(0.254,0.490) |
| g__[Ruminococcus] gnavus group | 0.30±0.83 | 0.60±1.24 | 2.198(0.028) | 0.365(0.249,0.482) |
| g__[Eubacterium] ventriosum group | 0.10±0.14 | 0.23±0.32 | 2.219(0.026) | 0.363(0.247,0.479) |
| g__Lachnospira | 0.12±0.25 | 0.26±0.56 | 2.267(0.023) | 0.360(0.242,0.478) |
| g__Tyzzerella 3 | 0.04±0.13 | 0.14±0.62 | 2.631(0.009) | 0.358(0.242,0.475) |
| g__Faecalibacterium | 5.83±9.17 | 9.92±10.14 | 2.395(0.017) | 0.352(0.236,0.468) |
| g__Subdoligranulum | 3.86±5.06 | 11.61±15.59 | 2.529(0.011) | 0.343(0.228,0.459) |
| g__Eggerthella | 0.04±0.11 | 0.08±0.17 | 2.607(0.009) | 0.343(0.228,0.459) |
| s__Enterococcus saccharolyticus subsp. saccharolyticus ATCC 43076 | 0.03±0.17 | 0.16±0.64 | 2.921(0.003) | 0.339(0.225,0.454) |
| g__Erysipelotrichaceae UCG-003 | 0.44±0.94 | 0.45±0.56 | 2.567(0.010) | 0.341(0.224,0.459) |
| g__Blautia | 2.39±2.14 | 3.44±2.49 | 2.650(0.008) | 0.336(0.222,0.450) |
| g__Flavonifractor | 0.05±0.10 | 0.34±0.98 | 2.768(0.006) | 0.329(0.217,0.442) |
| g__Lachnospiraceae NK4A136 group | 0.52±1.14 | 1.47±2.59 | 2.742(0.006) | 0.330(0.215,0.445) |
| g__[Eubacterium] hallii group | 1.24±2.24 | 1.69±1.75 | 2.804(0.005) | 0.326(0.211,0.442) |
| s__Asaccharobacter celatus | 0.06±0.18 | 0.27±0.47 | 2.933(0.003) | 0.323(0.207,0.439) |
| g__Ruminiclostridium 5 | 0.14±0.25 | 0.25±0.33 | 3.010(0.003) | 0.314(0.203,0.424) |
| g__Intestinibacter | 0.59±0.95 | 2.79±7.16 | 3.080(0.002) | 0.309(0.200,0.419) |
| s__Gordonibacter pamelaeae | 0.00±0.01 | 0.01±0.03 | 3.342(0.001) | 0.308(0.196,0.420) |
| g__Terrisporobacter | 0.03±0.07 | 0.20±0.67 | 3.395(0.001) | 0.293(0.184,0.403) |
| g__Anaerostipes | 0.36±1.07 | 0.85±1.01 | 3.556(0.000) | 0.280(0.171,0.389) |
| g__Romboutsia | 0.01±0.02 | 0.17±0.50 | 4.377(0.000) | 0.236(0.136,0.336) |
