# Supplementary material for: Fecal Microbiome Alteration May Be a Potential Marker for Gastric Cancer
Source: Dis Markers. 2020 Sep 15;2020:3461315. doi: 10.1155/2020/3461315 (PMC7519184; doi:10.1155/2020/3461315)
Supplement: Supplementary 4 — Table 4: ROC analysis of the different gut fungi between the GC patients and controls in the original population. [file 3461315.f4.docx]

**Table S4.** ROC analysis of the different gut fungi between the GC patients and controls in the original population

| The different fungal taxa | Relative abundance [(Mean±SD%)] | | *Z*(*P*) | AUC (95%CI) |
| --- | --- | --- | --- | --- |
|  | GC patients (n=134) | Control (n=58) |  |  |
| f__Pyronemataceae | 0.20±0.42 | 0.01±0.04 | 4.182(0.000) | 0.639(0.561,0.717) |
| f__Stachybotryaceae | 0.25±0.77 | 0.09±0.42 | 3.631(0.000) | 0.629(0.548,0.711) |
| g__Humicola | 0.76±1.55 | 0.11±0.24 | 3.298(0.001) | 0.629(0.549,0.708) |
| f__Lasiosphaeriaceae | 0.80±1.73 | 0.13±0.43 | 3.421(0.001) | 0.629(0.549,0.709) |
| s__Fusarium sp. | 0.48±1.22 | 0.06±0.21 | 2.918(0.004) | 0.623(0.540,0.705) |
| o__Auriculariales | 1.67±7.06 | 0.30±0.89 | 2.820(0.005) | 0.616(0.533,0.700) |
| s__Petriella sp. | 0.66±1.12 | 0.22±0.67 | 2.897(0.004) | 0.616(0.534,0.698) |
| g__Kazachstania | 0.17±0.66 | 0.01±0.05 | 3.322(0.001) | 0.604(0.523,0.685) |
| s__Aspergillus terreus | 0.29±0.84 | 0.00±0.00 | 3.664(0.000) | 0.601(0.519,0.682) |
| s__Sordaria sp. P44E2 | 0.22±0.65 | 0.00±0.02 | 3.033(0.002) | 0.596(0.514,0.678) |
| s__Cladosporium limoniforme | 0.18±0.50 | 0.02±0.13 | 3.233(0.001) | 0.595(0.513,0.678) |
| g__Chaetomium | 0.40±0.89 | 0.07±0.26 | 2.534(0.011) | 0.595(0.513,0.678) |
| s__Gibellulopsis nigrescens | 0.28±0.65 | 0.07±0.23 | 2.546(0.011) | 0.595(0.512,0.679) |
| s__Neurospora crassa | 0.30±0.76 | 0.06±0.39 | 2.817(0.005) | 0.594(0.511,0.676) |
| s__Trichoderma viride | 0.04±0.19 | 0.00±0.00 | 3.507(0.000) | 0.593(0.511,0.675) |
| g__Microascus | 0.05±0.15 | 0.00±0.00 | 3.507(0.000) | 0.593(0.511,0.675) |
| c__Leotiomycetes | 1.60±3.32 | 1.85±5.95 | 2.053(0.040) | 0.592(0.504,0.679) |
| f__Bionectriaceae | 0.30±1.01 | 0.05±0.19 | 2.555(0.011) | 0.591(0.508,0.674) |
| s__Aspergillus niger | 0.75±1.49 | 0.57±2.33 | 2.096(0.036) | 0.590(0.504,0.676) |
| s__Fusarium sp. GF8C2 | 0.15±0.42 | 0.01±0.04 | 2.782(0.005) | 0.590(0.507,0.673) |
| s__Thielavia sp. B27 | 1.52±2.78 | 0.69±1.72 | 2.106(0.035) | 0.590(0.506,0.673) |
| f__Sporormiaceae | 0.12±0.33 | 0.03±0.14 | 2.946(0.003) | 0.589(0.506,0.672) |
| g__Ascotricha | 0.03±0.14 | 0.11±0.32 | 3.255(0.001) | 0.408(0.316,0.500) |
| s__Ascosphaera apis | 0.31±1.34 | 0.32±0.79 | 2.715(0.007) | 0.403(0.314,0.493) |
| s__Kodamaea ohmeri | 0.71±5.95 | 0.69±3.26 | 3.257(0.001) | 0.399(0.308,0.490) |
| s__Vanrija fragicola | 0.17±0.56 | 0.53±1.18 | 2.914(0.004) | 0.394(0.303,0.486) |
| s__Aspergillus jensenii | 0.06±0.29 | 0.07±0.15 | 2.628(0.009) | 0.387(0.299,0.475) |
| s__Trichosporon coremiiforme | 0.09±0.19 | 0.47±2.02 | 2.784(0.005) | 0.385(0.297,0.473) |
| s__Aspergillus tabacinus | 0.08±0.35 | 0.11±0.41 | 2.844(0.004) | 0.377(0.292,0.462) |
| s__Cladosporium cladosporioides | 1.22±1.59 | 2.18±2.73 | 2.814(0.005) | 0.372(0.285,0.459) |
| s__Trichosporon asahii | 0.60±1.37 | 1.42±3.14 | 3.025(0.002) | 0.372(0.284,0.459) |
| s__Galactomyces candidum | 1.21±3.60 | 3.13±4.82 | 4.077(0.000) | 0.340(0.252,0.428) |
| s__Aspergillus sydowii | 1.14±4.18 | 3.29±11.77 | 3.558(0.000) | 0.339(0.256,0.421) |
| s__Cutaneotrichosporon cyanovorans | 0.30±0.88 | 0.56±0.96 | 3.903(0.000) | 0.335(0.251,0.420) |
| o__Wallemiales | 0.79±5.18 | 0.92±1.85 | 4.242(0.000) | 0.334(0.247,0.420) |
| s__Cutaneotrichosporon curvatus | 0.37±0.82 | 0.95±1.51 | 4.456(0.000) | 0.307(0.226,0.387) |
