# Supplementary material for: Fecal Microbiome Alteration May Be a Potential Marker for Gastric Cancer
Source: Dis Markers. 2020 Sep 15;2020:3461315. doi: 10.1155/2020/3461315 (PMC7519184; doi:10.1155/2020/3461315)
Supplement: Supplementary 5 — Table 5: ROC analysis of the different gut fungi between the GC patients and controls in the matching population. [file 3461315.f5.docx]

**Table S5.** ROC analysis of the different gut fungi between the GC patients and controls in the matching population

| The different fungal taxa | Relative abundance (Mean±SD%) | | *Z*(*P*) | AUC (95%CI) |
| --- | --- | --- | --- | --- |
|  | GC patients (n=44) | Control (n=44) |  |  |
| g__Humicola | 0.56±0.93 | 0.12±0.25 | 2.875(0.004) | 0.655(0.540±0.769) |
| f__Hypocreaceae | 0.58±1.13 | 0.11±0.27 | 2.782(0.005) | 0.653(0.539±0.768) |
| f__Stachybotryaceae | 0.20±0.41 | 0.12±0.47 | 3.015(0.003) | 0.639(0.522±0.757) |
| s__Petriella sp. | 0.73±1.11 | 0.24±0.74 | 2.513(0.012) | 0.634(0.517±0.751) |
| f__Lasiosphaeriaceae | 0.94±1.99 | 0.10±0.38 | 2.686(0.007) | 0.630(0.513±0.747) |
| f__Sporormiaceae | 0.18±0.46 | 0.03±0.16 | 2.907(0.004) | 0.624(0.506±0.742) |
| g__Chaetomium | 0.37±0.73 | 0.04±0.12 | 2.475(0.013) | 0.622(0.504±0.739) |
| s__Cutaneotrichosporon curvatus | 0.44±0.79 | 0.82±1.53 | 2.201(0.028) | 0.366(0.249±0.484) |
| g__Ascotricha | 0.01±0.06 | 0.13±0.36 | 3.605(0.000) | 0.354(0.238±0.470) |
| s__Cutaneotrichosporon cyanovorans | 0.32±0.90 | 0.65±1.03 | 2.635(0.008) | 0.342(0.227±0.456) |
